# Unclear association between levels of *Plasmodium falciparum* lactate dehydrogenase (*Pf*LDH) in saliva of malaria patients and blood parasitaemia: diagnostic implications?

**DOI:** 10.1186/s12936-017-2151-y

**Published:** 2018-01-05

**Authors:** Eva A. Nambati, William C. Kiarie, Francis Kimani, James H. Kimotho, Maureen S. Otinga, Edwin Too, Stephen Kaniaru, Janice Limson, Wallace Bulimo

**Affiliations:** 1 KEMRI Centre for Biotechnology Research and Development, Nairobi, Kenya; 2 Rhodes University Biotechnology Innovation Centre (RUBIC), Grahams Town, South Africa; 3 Department of Biochemistry, University of Nairobi, Nairobi, Kenya

**Keywords:** Non-invasive malaria diagnosis, Saliva, *Pf*LDH, Parasitaemia

## Abstract

**Background:**

The use of saliva in diagnosis of infectious diseases is an attractive alternative to procedures that involve blood drawing. It promises to reduce risks associated with accidental needle pricks and improve patient compliance particularly in malaria survey and drug efficacy studies. Quantification of parasitaemia is useful in establishing severity of disease and in assessing individual patient response to treatment. In current practice, microscopy is the recommended technique, despite its limitations. This study measured the levels of *Plasmodium falciparum* lactate dehydrogenase (*Pf*LDH) in saliva of malaria patients and investigated the relationship with blood parasitaemia.

**Methods:**

Matched pre-treatment blood and saliva samples were collected from patients at Msambweni District Hospital, Kenya. Parasitaemia was determined and only those confirmed to be *Plasmodium falciparum* mono-infected were recruited. *Pf*LDH was quantified in saliva using a commercial ELISA kit. A total of 175 samples were collected. Relationship between blood parasitaemia and concentration of *Pf*LDH in saliva was determined using Pearson correlation statistics. F test was used to determine whether there is a significant difference between levels of *Pf*LDH in saliva of patients with moderate to high parasitaemia and those with low parasitaemia.

**Results:**

One-hundred and seventy-five patient samples were positive for malaria by microscopy. Of these, 62 (35%) tested positive for *Pf*LDH in saliva, 113 (65%) were false negatives. For those that tested positive, (53) 85% were from patients with moderate to high parasitaemia while 9 (15%) were from patients with low parasitaemia. A correlation co-efficient of 0.18 indicated a weak positive relationship between the concentration of *Pf*LDH in saliva and blood parasitaemia. There was a marginal difference between levels of *Pf*LDH in saliva of patients with moderate to high parasitaemia and those with low parasitaemia [F (1, 59) = 1.83, p = 0.1807].

**Conclusion:**

The results indicate that there is a weak correlation between levels of *Pf*LDH in saliva and blood parasitaemia. This is weak association could be as a result of low sensitivity of the assay used as well as presence of inhibitors and proteases in saliva. Further studies should be focused towards reducing the number of false negatives and developing a customised assay that is specific for detection of *Pf*LDH in saliva.

## Background

Malaria continues to be a public health challenge particularly in resource-limited settings in sub-Saharan Africa, 3.3 billion people are at risk of developing the disease [1, 2]. Accurate diagnosis of malaria is a critical component in integrated approach for control and elimination of the disease [3]. Lack of diagnostic support particularly in field and resource-limited settings has led to over-reliance on clinical assessment. This practice has led to over-diagnosis of populations at risk posing a risk of rapid development of anti-malarial drug resistance [4, 5]. Microscopy remains the gold standard method for diagnosis of malaria. This is largely due to its ability of differentiate species and quantify parasitaemia. However, the technique is limited due to infrastructural and technical requirements that are not always available in resource-limited settings. In such settings, the adoption of malaria rapid diagnostic test (MRDT) has greatly improved access to diagnosis. However, its usefulness is limited due to its inability to quantify parasitaemia. In recent years, there have been significant efforts towards developing non-invasive malaria diagnostic tools. *Plasmodium falciparum* proteins HRP and LDH (P*f*LDH) have been detected in the saliva of malaria patients [6, 7]. Studies have demonstrated that levels of P*f*LDH in blood correlate with levels of parasitaemia [8, 9]. This is the first time P*f*LDH has been quantified in saliva of malaria patients and compared to parasitaemia. A positive correlation would provide a useful non-invasive assay that can be used to predict parasite load in malaria surveys of healthy populations, population-wide monitoring of the impact of anti-malarial interventions as well as in drug efficacy studies where repeated sampling of study cohorts is required.

The hypothesis was that there is a positive correlation between P*f*LDH concentration in saliva and blood parasitaemia which can be a useful tool in malaria studies.

## Methods

### Study site and enrolment

This study was conducted in Kwale County, Msambweni, Kenya, in a Kenya Medical Research Institute (KEMRI) established site at Msambweni District Hospital. This area is endemic to malaria with prevalence of 8%, as of the 2015 Kenya malaria indicator survey report [10]. Informed consent was obtained from the patients upon recruitment into the study.

### Initial screening

Patients aged between 2 and 65 years presenting with clinical symptoms of malaria were screened using RDTs (Malaria Ag Pf/Pan Standard diagnostics Inc).

### Diagnosis by microscopy

The infection status of patients found to be positive by RDT was confirmed by microscopy. At least two smears per patient were prepared and read by two independent microscopists. Six μl of blood was spread in a circular pattern of 12 mm diameter; 100 high-power fields of the thick film were examined to detect the presence of malaria parasites at 100 × oil immersion. Parasite density was calculated and recorded as number of parasites/μl blood using the following formula.

$$ {\text{Parasite density}} = {\text{Number of parasites counted}} \; \times \; {{{{ 8000{\text{ white blood cell}}} \mathord{\left/ {\vphantom {{ 8000{\text{ white blood cell}}} {\upmu{\text{l}}}}} \right. \kern-0pt} {\upmu{\text{l}}}}} \mathord{\left/ {\vphantom {{{{ 8000{\text{ white blood cell}}} \mathord{\left/ {\vphantom {{ 8000{\text{ white blood cell}}} {\upmu{\text{l}}}}} \right. \kern-0pt} {\upmu{\text{l}}}}} {{\text{Number of white blood cells counted}}.}}} \right. \kern-0pt} {{\text{Number of white blood cells counted}}.}} $$

where discrepancies were found a third reading was done. Only those found to be falciparum malaria (mono-infection) were recruited (Table 1).

**Table 1:** Sample quantification of P*f*LDH in saliva samples

Sample ID	Parasites/µl	OD	Concentration pg/ml
Positive blood sample	48,400	3.944	117,544.6
Saliva MSG 104	2560	0.055	Below detection limit
Saliva MSG 105	2160	0.087	Below detection limit
Saliva MSG 120	15,200	0.109	78.95249
Saliva MSG 041	38,800	0.1	69.472
Saliva MSG 035	32,320	0.101	70.46383
Saliva MSG 038	38,240	0.144	127.6701
Saliva MSG 078	32,400	0.145	129.3391
Saliva MSG 021	32,120	0.158	152.4354
Saliva MSG 116	40,300	0.131	107.3745
Saliva MSG 115	49,600	0.1480	134.4382
Saliva MSG 097	48,000	0.116	87.18796
Saliva MSG 042	53,600	0.146	131.0234
Saliva MSG 025	31,600	0.148	134.4382
Saliva MSG 106	4320	0.106	75.65383
Saliva MSG 026	2080	0.106	75.65383
Saliva MSG 124	1200	0.103	72.49367
Saliva MSG 147	6960	0.13	105.921
Saliva MSG 009	11,680	0.065	Below detection limit
Saliva MSG 104	2560	0.082	Below detection limit
Saliva MSG 015	39,600	0.074	Below detection limit
Negative saliva sample	–	0.053	N/A
Negative control (kit)	–	0.066	N/A

Blood sample with parasitaemia of 48,400 had a concentration of 117,544.6 pg/ml while saliva MSG 115 and MSG 097 with similar parasitaemia range had 134.4382 pg/ml and 87.18796 pg/ml, respectively. False negatives were present in samples with low, moderate as well as high parasitaemia MSG 104 and MSG 015, respectively. The ELISA results for saliva samples were generally weak positives compared to blood samples

### Collection of saliva samples

A matched saliva sample was collected from patients confirmed to be falciparum malaria (mono-infection). Saliva samples were collected using saliva collection aid (Salimetrics, USA) and immediately stored at − 80 °C without processing. Patients were instructed to allow saliva to passively flow into the saliva collection aid; approximately 1 ml of saliva was collected from each patient.

### *Plasmodium falciparum* LDH enzyme-linked immunosorbent assay (*Pf*LDH ELISA)

*Pf*LDH in saliva was detected and quantified using a commercial malaria antigen ELISA kit (Standard Diagnostics Inc). The kit is designed for qualitative detection of malaria *Plasmodium* species PLDH antigen in human whole blood and has a sensitivity of 98% for *P. falciparum*. The ELISA assays were run as per manufacturer’s instructions. Prior to analysis, the samples were thawed on ice. Fifty μl of each test sample (saliva) was mixed with 50 μl of phosphate-buffered saline supplemented with 0.05% Tween 20 (PBS/T) and allowed to react for 15 min with 100 μl of PLDH detector antibody solution. The reaction mixture was transferred into the ELISA test plate, pre-coated with the capture antibody provided in the kits. The plates were incubated for 60 min at 37 °C to allow effective binding of the analyte sandwiched between the corresponding capture and detector antibodies. The wells were washed and 100 μl chromogen (TMB) substrate, supplied in the kit, was added. The relative absorbance values were read using a microplate reader at 450 nm. The amount of bound sandwiched target analyte was measured as optical density (OD). The cut-off value was 0.1 and was determined as per manufacturer’s instructions (Table 1).

### Comparison of *PfLDH* levels in saliva and blood

The difference in concentration of *Pf*LDH in saliva and blood was determined using a commercial malaria antigen ELISA kit (Standard Diagnostics Inc). The ELISA assays were run as per the manufacturer’s instructions. Prior to analysis the blood and saliva samples were thawed on ice. The ELISA assay was run as previously described. Negative samples of patients confirmed to be negative for malaria were also included in the ELISA assay (Table 1).

### Statistical analysis

The F test was used to determine whether there is a significant difference between levels of *Pf*LDH in saliva of patients with moderate to high parasitaemia > 1000 parasite/μl and those with low parasitaemia < 1000 parasites/μl. A comparison between the *Pf*LDH ELISA and parasite density was done using Pearson correlation statistics.

## Results

### Detection and quantification of *Pf*LDH in saliva of malaria patients

A total of 175 patients were confirmed to be *P. falciparum*-positive by microscopy and recruited into the study. Thirty-five per cent of the patients recruited into study tested positive for *Pf*LDH ELISA in saliva. The remaining 65% were false negatives.

**Fig. 1:**
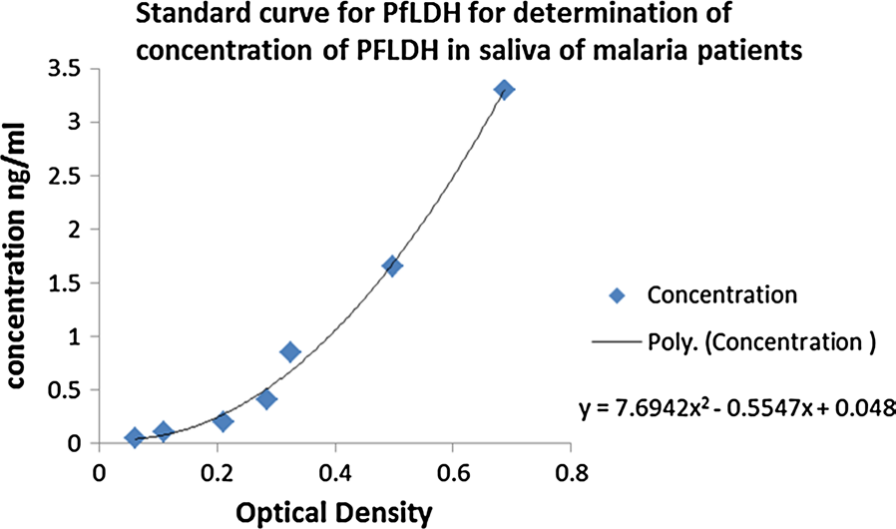
Standard curve for determination of concentration of PfLDH in saliva of malaria patients. The standard curve was calculated and the standard fitted using Excel non-linear regression. The concentration of the P*f*LDH in patient saliva was then estimated from the standard curve

The concentration of *Pf*LDH in saliva of malaria patients was several folds lower than the concentration of *Pf*LDH in blood of malaria patients with similar range of parasitaemia. The occurrence of false negatives was observed in both low and high parasitaemia samples. The ELISA assay generally gave weak positives for P*f*LDH in saliva (Fig. 1).

### Association between levels of blood parasitaemia and *Pf*LDH levels in saliva of malaria patients

**Fig. 2:**
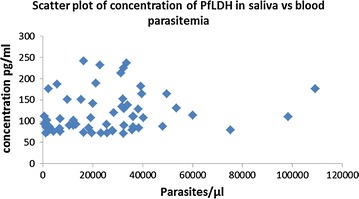
Scatter plot of concentration of PfLDH in saliva vs blood parasitaemia. The concentration of P*f*LDH in saliva was plotted against the matched parasites/μl. Pearson correlation was used to determine the relationship between concentration of PfLDH in saliva and blood parasitaemia

A correlation coefficient of 0.18 indicated a slight linear positive relationship between the concentration of *Pf*LDH in saliva and parasitaemia in blood. There was a slight difference between levels of *Pf*LDH in saliva of patients with moderate to high parasitaemia and those with low parasitaemia [F (1, 59) = 1.83, p = 0.1807] (Fig. 2).

## Discussion

The use of saliva as an alternative non-invasive diagnostic matrix for infectious diseases continues to elicit interest. However, despite numerous reports on the diagnostic potential of saliva, there is still a limited number of saliva-based assays in use in clinical settings compared to its plasma counterparts. This can be attributed partly to the fact that the target analytes are usually present in many folds higher concentration in blood that in saliva, making plasma-based assays more sensitive. This has limited the development of saliva-based, malaria, point-of-care diagnostic tools. However, saliva remains an attractive alternative for diagnosis as it is less bio-hazardous, non-invasive and relatively easy to collect from patients. Compliance to blood drawing may not be an issue for malaria patients in clinical settings, however, an investigator may face challenges convincing healthy patients in a malaria survey study or drug efficacy study to comply with multiple blood drawing. It is thus worthwhile to further advance the quest for saliva diagnostics. This study sought to investigate the potential use of saliva in predicting parasite load by investigating the relationship between blood parasitaemia and concentration of P*f*LDH in saliva of malaria patients. A positive correlation would provide a useful diagnostic tool that can be used to enhance malaria surveys studies as well as drug efficacy studies.

### P*f*LDH in malaria

*Plasmodium* lactate dehydrogenase (PLDH) is an intracellular glycolytic enzyme that catalyses oxidation of lactate to pyruvate. Each *Plasmodium* species has a variant isomer of the enzyme. The enzyme is expressed in high levels during the asexual stage of the parasites and clears within 24 h of effective malaria treatment [11]. P*f*LDH was selected for this study based on studies that have demonstrated its presence in saliva [7] and its correlation with the parasite density in plasma of malaria patients [11, 12]. The measurement of concentration of P*f*LDH in saliva of malaria patients and relating it to parasitaemia could offer an alternative non-invasive assay that can be used to predict parasite load and potentially be used in assessing patient response to treatment. This paper reports for the first time the detection and quantification of P*f*LDH in saliva of malaria patients on day 0 prior to treatment and its correlation to parasitaemia.

A significant number of false negatives were observed and there were variations in concentration of P*f*LDH in individuals with comparable parasitaemia. A weak positive correlation between lactate dehydrogenase concentration in saliva and parasite density was observed. There was a marginal difference between levels of *Pf*LDH in saliva of patients with moderate to high parasitaemia and those with low parasitaemia.

In the current study a commercial kit Malaria Antigen ELISA kit (Standard Diagnostics Inc) was used to detect P*f*LDH in saliva. Other studies have reported qualitative detection of *Plasmodium* antigen HRP in saliva using commercial kits designed for detection of *Plasmodium* antigens in plasma. However, the detection was limited due to requirement of low detection levels when it comes to saliva samples [9]. Detection of PLDH in saliva of malaria patients using RDTs has also been reported [7]. This is the first time P*f*LDH in saliva of malaria patients has been detected and quantified using ELISA. Other studies have reported improved detection of *Plasmodium* antigens (HRP) in saliva when a customized in-house ELISA assay is developed [9]. This study further corroborates the viability of saliva in malaria diagnostics. The assay was limited to the limit of detection (LOD) of a commercial kit designed for use with plasma samples. This could partly explain the occurrence of false negatives. Variations in concentration of P*f*LDH in saliva of individuals with similar ranges of parasitaemia suggests that there are inherent factors in saliva that affect the assay [13, 14]. The stability of PLDH in saliva is not equivalent to its stability in plasma. Saliva has proteases that may contribute to the breakdown of the enzyme (PLDH) in saliva. Noteworthy is the clarity of the saliva samples which varied between individuals despite the request for patients to give unstimulated drool; it was also difficult to get clear saliva samples from children who were dehydrated. Future work should be focused on standardizing saliva collection as well and standardization of saliva samples prior to running the ELISA assay. In addition the development of an in-house ELISA assay for the detection of PLDH in saliva samples may also help in reducing the number of false negatives.

## Conclusion

This study has demonstrated that there is a weak correlation between the levels of P*f*LDH in saliva and blood parasitaemia in malaria-positive patients. For the assay to be useful, the sensitivity of the test has to be improved. In this study, the sensitivity was limited due to use of a commercial assay designed for plasma samples. Future work should be focused on the development of a customized saliva assay that would take into consideration the complex saliva matrix and mitigate the effects of proteases and mucins that may have contributed to low sensitivity.
